# FMR1 deletion in rats induces hyperactivity with no changes in striatal dopamine transporter availability

**DOI:** 10.1038/s41598-022-26986-2

**Published:** 2022-12-29

**Authors:** Annunziata D’Elia, Sara Schiavi, Antonia Manduca, Alessandro Rava, Valeria Buzzelli, Fabrizio Ascone, Tiziana Orsini, Sabrina Putti, Andrea Soluri, Filippo Galli, Alessandro Soluri, Maurizio Mattei, Rosella Cicconi, Roberto Massari, Viviana Trezza

**Affiliations:** 1 Institute of Biochemistry and Cell Biology (IBBC), National Research Council of Italy (CNR), c/o International Campus “A. Buzzati-Traverso”, Via E. Ramarini, 32, 00015 Monterotondo Scalo (Rome), Italy; 2 Department of Science, Section of Biomedical Sciences and Technologies, Roma Tre University, Viale G. Marconi 446, 00146 Rome, Italy; 3 Neuroendocrinology, Metabolism and Neuropharmacology Unit, IRCSS Fondazione Santa Lucia, Rome, Italy; 4 Unit of Molecular Neurosciences, University Campus Bio-Medico, Rome, Rome, Italy; 5 Nuclear Medicine Unit, Department of Medical-Surgical Sciences and of Translational Medicine, Faculty of Medicine and Psychology, “Sapienza” University of Rome, Rome, Italy; 6 Department of Biology and Centro di Servizi Interdipartimentale-Stazione per la Tecnologia Animale, “Tor Vergata” University, Rome, Italy

**Keywords:** Motor control, Social behaviour, Psychiatric disorders, Imaging, Molecular imaging, Positron-emission tomography, Biological techniques, Neuroscience

## Abstract

Autism Spectrum Disorder (ASD) is a pervasive neurodevelopmental disorder emerging in early life characterized by impairments in social interaction, poor verbal and non-verbal communication, and repetitive patterns of behaviors. Among the best-known genetic risk factors for ASD, there are mutations causing the loss of the Fragile X Messenger Ribonucleoprotein 1 (FMRP) leading to Fragile X syndrome (FXS), a common form of inherited intellectual disability and the leading monogenic cause of ASD. Being a pivotal regulator of motor activity, motivation, attention, and reward processing, dopaminergic neurotransmission has a key role in several neuropsychiatric disorders, including ASD. *Fmr1* ^*Δ*^*exon 8* rats have been validated as a genetic model of ASD based on FMR1 deletion, and they are also a rat model of FXS. Here, we performed behavioral, biochemical and in vivo SPECT neuroimaging experiments to investigate whether *Fmr1* ^*Δ*^*exon 8* rats display ASD-like repetitive behaviors associated with changes in striatal dopamine transporter (DAT) availability assessed through in vivo SPECT neuroimaging. At the behavioral level, *Fmr1* ^*Δ*^*exon 8* rats displayed hyperactivity in the open field test in the absence of repetitive behaviors in the hole board test. However, these behavioral alterations were not associated with changes in striatal DAT availability as assessed by non-invasive in vivo SPECT and Western blot analyses.

## Introduction

The 5th Edition of the Diagnostic and Statistical Manual of Mental Disorders^1^ defines autism spectrum disorder (ASD) as a neurodevelopmental disorder characterized by persistent deficits in social communication and interaction and restricted-repetitive patterns of behavior, interests or activities. Fragile X syndrome (FXS) is a common form of inherited intellectual disability (ID) and the leading monogenic cause of ASD^2,3^. It is most commonly caused by a trinucleotide repeat expansion of CGG in the promoter region of Fragile X Messenger Ribonucleoprotein 1 (FMR1) gene, leading to methylation, transcriptional silencing and to the absence or deficiency of FMRP. FMRP is an RNA binding protein with a key role in the translational control of several mRNAs, many of which are involved in the maintenance and development of synaptic function and plasticity^4^. Therefore, in the absence of this protein, deregulation of translation, transport, and mRNA stability affects multiple neuronal pathways, generating the characteristic phenotype of FXS patients. Approximately 30% of patients with FXS meet the full diagnostic criteria for ASD^5^, and over 90% of individuals with FXS display some ASD symptoms^6^, including cognitive deficits, social dysfunctions, mood lability, hyperactivity, altered sensory processing and seizures.

Dopaminergic neurotransmission is a critical regulator of motor function, reward, motivation, attention and learning^7–10^. Dysregulations of the brain dopaminergic system have been implicated in a number of neurological and neuropsychiatric disorders, including ASD^11–13^. Interestingly, the dopamine (DA) hypothesis of ASD claims that dysfunctions in the midbrain dopaminergic system could contribute to autistic-like behaviors^14^: thus, the social deficits observed in ASD could reflect a mesocorticolimbic circuit dysfunction, while the repetitive/stereotyped behaviors could arise from a dysfunction of the nigrostriatal pathway^15^. Dopaminergic projections originating from the substantia nigra and ventral tegmental area terminate in the striatum, where they regulate motor function and overall activity and influence thalamocortical signaling^16^. The striatal complex of basal ganglia comprises two functionally distinct districts, the dorsal and ventral striatum, with the former mainly involved in the control of motor activities together with procedural memory storage, and the latter principally mediating motivation, reward, and emotion^17^. Due to the prominent role of the striatum in sensorimotor function (e.g., locomotor control and habit formation), associative tasks (e.g., goal-directed behavior) and motivational behavior^18–20^ and its abundance in DA projections^21^, it is possible that aberrant DA striatal signaling could promote the stereotyped and perseverative patterns of behaviors typical of ASD^22,23^.

The DA transporter (DAT) plays a fundamental role in maintaining optimal DA signaling, since it regulates the temporal and spatial availability of DA^24^ by rapidly clearing released DA from the synapse. Interestingly, DAT gene mutations have been linked to ASD^12^, and altered DAT expression in rodents has been correlated to hyperactivity and repetitive behaviors^25,26^, which are hallmarks of ASD^27,28^. Though these studies have been incremental to the field, our understanding of the impact of DAT dysregulation in the behavioral deficits typically associated with ASD and its comorbidities is still limited.

*Fmr1-*^*Δ*^*exon 8* rats have been recently validated as a genetic animal model of ASD and rat model of FXS^29^. Interestingly, the *Fmr1-*^*Δ*^*exon 8* rat model—generated by zinc-finger nucleases (ZFN)—results in a gene product with a loss of exon 8 which encodes a domain within the FMR1 gene that is responsible for RNA-binding, the KH1 domain. Although this animal model differs in the type of mutation from humans, this deletion is sufficient to cause FXS-like traits. In line with this, we have recently shown that *Fmr1-*^*Δ*^*exon 8* rats show cognitive, communicative and social impairments^30,31^, suggesting the validity of this animal model in mimicking the key behavioral deficits that characterize FXS and some of the core and comorbid features of non-syndromic ASD. Here, we investigated whether *Fmr1-*^*Δ*^*exon 8* rats show altered motor function eventually associated with changes in striatal DAT availability.

During the last decade, high-resolution positron emission tomography (PET) and  single-photon emission computed tomography (SPECT) scanners have been increasingly employed to study neurotransmitter transporter and receptor binding in small laboratory animals, giving new insights into the involvement of dopaminergic neurotransmission in neurological and psychiatric disorders^32–35^. Indeed, while other tools have been used in the preclinical field to assess DAT function, some techniques are either invasive or ex vivo techniques that do not allow longitudinal studies. Conversely, the use of a neuroimaging technique such as SPECT allows the correlation between behavioral outputs and the activation/inactivation of a specific molecular target in a given brain region. Upon injection of the radiopharmaceutical, SPECT freezes a snapshot of brain activity, which might be then recorded and analyzed. Additionally, while other techniques, such as chronoamperometry or voltammetry, may provide better temporal and spatial resolutions, SPECT provides not only quantification but also the visualization of data in their anatomical reference through its simple integration with other morphological imaging systems. Furthermore, it is a relatively simple technique that causes minimal stress to the animal by being non-invasive, and it has the potential of enabling longitudinal studies. To allow translational research, several dedicated imaging systems for small laboratory animals, such as mice and rats, have been developed over the years. Nevertheless, the extension of imaging modalities from humans to small animals faces some limitations mainly related to the differences in size and intensity of biochemical signals between the two species. For a better multidimensional understanding of complex pathophysiological phenomena, the real current challenge would be to design increasingly sensitive systems with high spatial resolution. In this context, our group has recently developed an innovative SPECT system for small organ imaging and neuroimaging that implements a super spatial resolution (SSR) method to exceed imaging capabilities achievable with traditional systems^36–39^. This system offers the unique opportunity to improve achievable performance with the potential to obtain higher-quality preclinical functional brain imaging^39^. This is achieved by moving the detector with sub-pixel shifts. In this way, the counting information of the pixel area is virtually divided into smaller areas, thus increasing the resolution of the images. The exploitation of the SSR algorithm for scintigraphic applications represents a breakthrough that might improve the performance of new generation SPECT scanners. Taking advantage of this technique, we combined behavioral and in vivo SPECT neuroimaging analyses as a preliminary investigation of the role of striatal DAT in the dysfunctional motor behavior of *Fmr1-*^*Δ*^*exon 8* rats. Exploring the neurobiological mechanisms involved in repetitive behavior and motor impairments in ASD and co-occurring conditions including FXS will improve our understanding of the pathogenesis of these developmental neuropsychiatric disorders, stimulating research on novel therapeutic approaches to these conditions.

## Methods

### Experimental design

**Figure 1:**
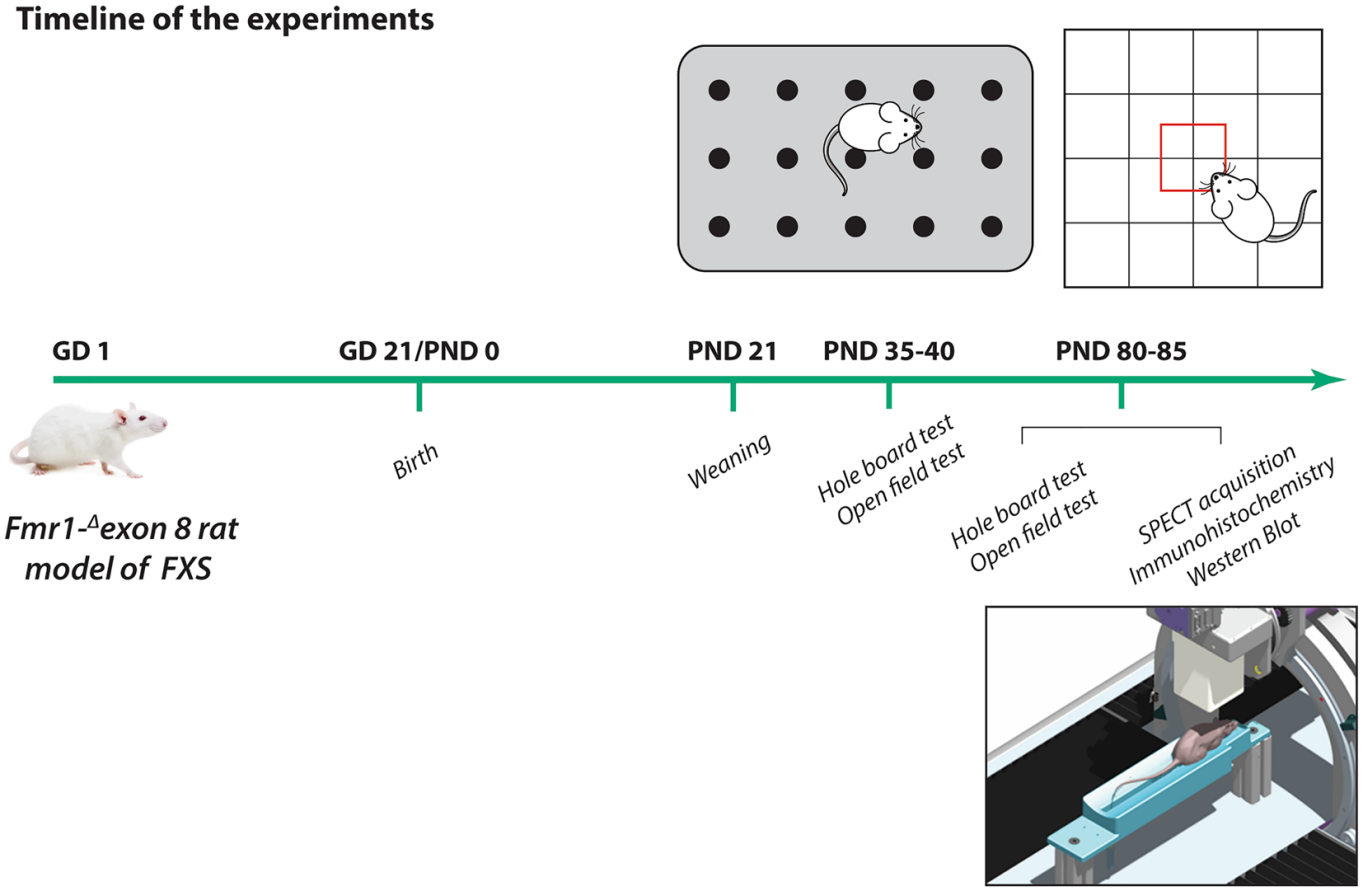
Timeline of the experiments.

Control (wild-type) and *Fmr1-*^*Δ*^*exon 8* rats were used to perform the experiments described below. The behavioral experiments were performed in juvenile (35–40 days old) and adult (80–85 days old) rats to evaluate locomotor and potential stereotypic behavior across development. Western blot experiments, histological assay and imaging studies were conducted in adult rats. The experimental design is shown in Fig. 1.

### Animals

Wild-type (WT) (Charles River Laboratories, Italy) and *Fmr1-*^*Δ*^*exon 8* male and female rats (Horizon Discovery, formerly SAGE Labs, USA) on a Sprague–Dawley background were mated overnight. Pregnant rats were individually housed in Macrolon cages (40 (length) × 26 (width) × 20 (height) cm), under controlled conditions (temperature 20–21 °C, 55–65% relative humidity, and 12/12 h light cycle with lights on at 07:00 h). Newborn litters found up to 17:00 h were considered to be born on that day (postnatal day (PND) 0). On PND 1, the litters were culled to eight animals (six males and two females), to reduce any litter size-induced variability in the growth and development of pups during the postnatal period. On PND 21, the pups were weaned and housed in groups of three (same-sex and same genotype) and tested across development. One pup per litter from different litters per treatment group was randomly used in each experiment. The experiments were conducted on the male offspring. The experiments were approved by the Italian Ministry of Health (Rome, Italy; Authorization N° 849/2020-PR) and performed in agreement with the ARRIVE (Animals in Research: Reporting In Vivo Experiments) guidelines^40^, the guidelines of the Italian Ministry of Health (D.L. 26/14) and the European Community Directive 2010/63/EU.

### SPECT System

The High-Resolution Imaging System (HiRIS2) for preclinical studies has been used to quantify the dopamine transporter (DAT) binding in the brain of the *Fmr1-*^*Δ*^*exon 8* and WT rats using [^123^I]FP-CIT as radioligand. The dedicated SPECT scanner consists of two detection heads housed in a rotating gantry. The experimental subject is placed on an animal bed that can move axially with respect to the rotation plane. The mechanics are designed to allow the necessary movements of the detectors for the application of the Super Spatial Resolution (SSR) technique which could be applied to improve the effective spatial resolution achievable, thus representing a key element for the study of very small brain structures^38^. To this purpose, multi-degree freedom carriages are used to obtain a fine alignment both linear and planar of the detectors. Specifically, each HiRIS2 head is based on an H13700 Hamamatsu PSPMT coupled to a CRY018 pixelated scintillator and a low-energy tungsten collimator with parallel square holes. Finally, to obtain a 3D SPECT imaging, the two detectors were mounted in opposition at 180° from each other.

### Behavioral procedures

The animals were habituated to the experimental room before testing. To minimize stress responses and to allow the animals to familiarize with the operator, rats were extensively handled for few consecutive days before testing by the same operator who performed the test. Notably, the scoring-designed operator was not the same operator who manipulated the animals and performed the test, and was unaware of animals' genotype; in other words, scoring was done in blind conditions.

#### Hole board test

The test was performed in a sound-attenuated chamber under dim light conditions, as previously described^41–44^. The apparatus consisted of a grey square metal table (40 × 40 × 10 cm; l × w × h) with 16 evenly spaced holes (4 cm in diameter), inserted in a Plexiglas arena (40 × 40 × 60 cm; l × w × h). Each rat was individually placed in the apparatus for 5 min. Each session was recorded with a camera positioned above the apparatus for subsequent behavioral analysis performed using the Observer 3.0 software (Noldus Information Technology, NL). Dipping behavior was scored as the number of times an animal inserted its head into a hole at least up to the eye level.

#### Open field test

The test was performed as previously described^45^. The apparatus consisted of a Plexiglas arena 45 × 45 cm, illuminated by fluorescent bulbs at a height of 2 m above the floor of the open field apparatus (light intensity of 30 lx). The floor was cleaned between each trial to avoid olfactory clues. Each animal was transferred to the open field facing a corner and was allowed to freely explore the experimental arena for 15 min. The locomotor activity was scored as follows^43^: a grid, dividing the arena into equally sized squares, was projected over the recordings, and the number of line crossings made by the animal (i.e., the frequency of the animal's passage from one section of the grid to another) was recorded using the Observer 3.0 software (Noldus Information Technology, NL). Notably, the crossing was counted at the time when the animal passed from one section to another with all four paws^46^. Spontaneous rearing behaviors, in which rodents stand on their hind legs with the intention of exploring, were also counted during the 15-min test period. We defined two forms of rearing: unsupported rearing (in which the animal rears without contacting the walls of the arena) and wall rearing (in which the animal rears against the walls of the arena). To evaluate thigmotaxis (i.e., the animal remains closely in proximity to the walls of the open field), the time spent at periphery and at center of the open field was also measured and reported as percentage of the total time.

#### Elevated Plus Maze test

The elevated plus maze apparatus comprised two open (50 × 10 × 40 cm; l × w × h) and two closed arms (50 × 10 × 40 cm; l × w × h) that extended from a common central platform (10 × 10 cm). The test was performed as previously described^47,48^. Rats were individually placed on the central platform of the maze for 5 min. Each 5-min session was recorded with a camera positioned above the apparatus for subsequent behavioral analysis carried out an observer, unaware of animal treatment, using the Observer 3.0 software (Noldus Information Technology, NL). The following parameters were analyzed:

- % Time spent in the open arms (% TO): (seconds spent on the open arms of the maze/300 s) × 100;
- % Open arm entries (% OE): (the number of entries into the open arms of the maze/number of entries into open + closed arms) × 100;
- Number of head dipping (downward movement of rodents’ head toward the floor from the open arms);
- Number of total entries (frequency of entries into closed and open arms and the center of the maze).

### Neuroimaging studies

SPECT measurements of DAT binding sites were performed with the HiRIS2 SPECT system as previously described^39^. Animals were pre-treated by oral gavage with 10 μl of Lugol’s solution, a thyroid blocking agent, 1 h prior to the administration of the radiopharmaceutical. This will decrease the radiation dose to the thyroid and preclude possible adverse effects on the gland, while also allowing better brain imaging by concentrating iodine accumulation on the targeted area^49–51^. Then, the animals were anesthetized using isoflurane (IsoFlo, Zoetis, UK) at a concentration of 3% for induction and 2% for maintenance. In vivo DAT binding was measured using Methyl (3S,4S,5R)-8-(3-fluoropropyl)-3-(4-iodophenyl)-8-azabicyclo[3.2.1]octane-4-carboxylate([^123^I]FP-CIT, DaTSCAN^®^) as radioligand, since it is widely used to assess the pre-synaptic striatal uptake in the basal ganglia of the rat brain^52–55^. A dose of 37 ± 4 MBq in 1,25 ml DaTSCAN (GE Healthcare, DE) was administered into the lateral tail vein. Imaging measurements were started 2 h after radioligand administration when the equilibrium post-injection of [^123^I]FP-CIT binding is reached, with the ratio of specific to non-specific striatal uptake remaining stable over the following 4 h^56^.

Generally, in small animal imaging studies intravenous injection of [^123^I]FP-CIT (DaTSCAN) has been used to visualize both the DAT and the serotonin transporters (SERT)^54^. However, DaTSCAN injection results in high radioactivity accumulation in the striatum. In comparison, less pronounced uptake has been observed in brain areas with a high density of serotonergic uptake sites, such as the midbrain^56^. Therefore, when assessing striatal DAT function in vivo in laboratory animals, only striatal signal is usually considered, both because of the limited sensitivity and resolution of the available instrumentation and because of the possibility of seeing elevated extra-striatal signals in the midbrain also due to the binding of DaTSCAN to the SERT^57,58^.

Both planar and SPECT acquisitions were performed. Planar images were obtained by 30 min acquisitions. Whereas, each SPECT scan was carried out by collecting 48 angular projections over an arc of 360° (24 steps of 7.5°). Since the SSR method was applied, two images per angular position were acquired. Subsequently, the total scan time was 48 min. Since the CT module is not implemented on the HiRIS2 prototype, we could perform a CT scan on each rat’s brain using a U-CT preclinical system (MILabs B.V., NL). SPECT reconstruction was performed with the iterative ordered-subset-expectation–maximization (OSEM) algorithm using a priori knowledge of the CT data providing a precise localization of the radiotracer uptake.

### Computed Tomography (CT) imaging

For coregistration of SPECT functional data to tomographic ones, three-dimensional morphological volume measurements were acquired with a micro-CT scanner having 0.08 mm isotropic resolution. The rats' brains of both *Fmr1-*^*Δ*^*exon 8* rats and their WT control group were scanned using a MILabs 3D optical CT scanner (MILabs B.V., NL). CT scanner settings were identical for all scans. Each rat brain was 3-dimensionally (3D) micro-CT acquired at 80-μm voxel size resolution using the following settings: 720 steps of 0,5°; exposure 40 ms; voltage 50 kV; current, 0.43 mA, yielding a total scan time of about 2,5 min. The reconstruction output was the Neuroimaging Informatics Technology Initiative (NIftI) file type, which is a format commonly used in preclinical nuclear imaging informatics. CT scan reconstructions were performed using filtered back-projection (FBP) for the registration to SPECT scans. Finally, coregistered images were processed using Mango—Multi-image Analysis GUI (Research Imaging Institute, UTHSCSA)^59^, and AMIDE (Amide's a Medical Image Data Examiner)^60^ image software. Axial, coronal, and sagittal views and SPECT slices co-registered with the anatomic reference provided by the CT are shown in Fig. 5.

### Histology

At the end of the experiments, the animals were sacrificed, and brain samples were quickly collected and fixed in formalin 10%. For hematoxylin (Sigma-Aldrich, cat. MHS16) and eosin (Sigma-Aldrich, cat. 109,844) histology assay samples were paraffin embedded. Microtome-sectioning was conducted to generate 8-μm sections that were deparaffinized and rehydrated. For histological analysis, standard hematoxylin and eosin staining was performed to evaluate significant changes in the gross tissue organization of the dorsal striatum between *Fmr1-*^*Δ*^*exon 8* and WT rats, and bright-field images were acquired using an inverted microscope equipped with 6 × and 40 × objective (Leica Microdissector—LMD 7000, camera Leica DFC310 FX). The histological and immunostaining assays were performed on dorsal striatum to further support the results achieved by SPECT analysis.

### Immunofluorescence

Immunofluorescence was conducted on deparaffinized and rehydrated 8-μm sections. Antigen retrieval was conducted with citrate buffer pH-6, boiling solution for 10 min. Permeabilization was carried out with 0.25% Triton X-100 (Sigma-Aldrich, cat. 9002-93-1) in TBS 1X with 5% goat serum (Sigma-Aldrich, cat. G9023) for 1 h at room temperature (RT). Samples were incubated overnight in a humidified chamber at 4 °C with primary antibody anti-DAT (1:50, 22,524-1AP, Rabbit Polyclonal, Proteintech), in blocking buffer (TBS 5% goat serum). The day after, slides were washed with TBS 1X supplemented with 0.2% Tween-20 (Sigma-Aldrich, cat. P1379) and incubated with cross-adsorbed Alexa Fluor 568 secondary antibody (goat-anti-rabbit IgG A11011, Invitrogen) for 1 h at RT, then washed again and stained with DAPI (5 μM in PBS 1X for 5 min). The slides were covered with prolong (P36930 ProLong™ Gold Antifade Mountant, Thermo Fisher) and closed with cover slips (Menzel-Glaser, Thermo Fisher). Fluorescence images were acquired using an inverted microscope equipped with 40 × objective (Leica Microdissector—LMD 7000, camera Leica DFC345 FX).

### Immunohistochemistry

Immunohistochemistry was conducted on deparaffinized and rehydrated 8-μm sections. Antigen retrieval was conducted with citrate buffer pH-6 boiling solution for 10 min. To block endogenous peroxidase activity, we used an incubation for 30 min in a solution of TBS-T 1% hydrogen peroxide final. Permeabilization was carried out with 0.25% Triton X-100 (Sigma-Aldrich, cat. 9002-93-1) in TBS 1X with 5% goat serum (Sigma-Aldrich, cat. G9023) for 1 h at RT. Samples were incubated overnight in a humidified chamber at 4 °C with primary antibody anti-DAT (1:50, 22,524-1AP, Rabbit Polyclonal, Proteintech), in blocking buffer (TBS 5% goat serum). The next day, slides were washed with TBS 1X (Bio-Rad, cat. 1,706,435) 0.2% TWEEN-20 (Sigma-Aldrich, cat. P1379) and incubated with anti-rabbit secondary antibody (Vectastain, Kit PK-4001). Finally, we used ABC Kit (Vectastain, PK-6100) as indicated, to amplify the signal. The substrate used to reveal staining was DAB (Sigma-Aldrich, cat. D5905) and the reaction was blocked with 1X PBS 5 mM EDTA. After dehydration, slides were covered with mounting media Entellan (Merck, cat. 1,079,600,500). Bright-field images were acquired using an inverted microscope equipped with 6 × objective (Leica Microdissector—LMD 7000, camera Leica DFC310 FX). All the histological and immunostaining assays were performed on the dorsal striatum of adult *Fmr1-*^*Δ*^*exon 8* and WT rats.

### Evaluation of Neuroimaging studies

Regarding planar acquisitions, for each rat, SPECT and CT images were coregistered within the aim of the Paxinos standard rat brain MRI46 provided by the Neuroimaging Tools & Resources Collaboratory (NITRC Image Repository (NITRC-IR)—www.nitrc.org). At this point, image processing and analysis were performed through a semi-quantitative evaluation of the region of interest (ROI) on brain-activated areas. In particular, as shown in Fig. 3, imaging data were assessed by defining an area comprising each striatum, and another corresponding to the cerebellum region. Thus, maximum striatal count rates (counts/pixel), as well as cerebellar reference count rates (counts/pixel), were determined. Left and right striatal counts rates were averaged. Semi-quantitative measures for the quantification of the binding potential were assessed as ratios between the striatal specific uptake and the non-specific cerebellum uptake^61^. Particularly, the striatal specific binding ratio (SBR_STR_) was calculated as:

$$SBR_{STR} = \frac{{C_{STR} - C_{CB} }}{{C_{CB} }}$$

where C_STR_ and C_CB_ are, respectively, the mean count in the striatal and in the cerebellum ROI. Instead of the volumetric reconstructions, three-dimensional CT and all reconstructed SPECT studies were transferred to a dual Xeon processor (Intel Corporation, USA) workstation for images analysis and 3D rendering (Fig. 5).

### Western blot analysis

To complement the SPECT and immunohistological findings, we performed Western blot experiments to evaluate DAT protein levels in both the dorsal and ventral striatum of *Fmr1-*^*Δ*^*exon 8* rats compared to WT control animals. Tissue samples were collected and stored at -80 °C. Dorsal and ventral striatum were sonicated in ice-cold RIPA buffer supplemented with protease (Roche, cat. 05,892,791,001) and phosphatase (Roche, cat. 4,906,845,001) inhibitors, centrifuged at 13.000 rpm for 15 min and supernatants were collected. Protein content was quantified by using the colorimetric Breadford assay and 15 μg of proteins for each sample were loaded and resolved through SDS-PAGE electrophoresis at 120 V. Subsequently, proteins were transferred on a nitrocellulose membrane by Turbo-blot system (2.5 V, 25 mA, 3 min), and a Ponceau staining was performed to verify the quality of transfer. Membrane was then blocked in everyblot blocking buffer (Bio-Rad, cat. 12,010,020) for 5 min and incubated with the primary antibody rabbit anti-DAT (1:2000, Proteintech, cat. 22,524–1-AP) in TBS with 3% BSA + 0.1% Tween-20 overnight at + 4 °C. After washings, membranes were incubated with horseradish peroxidase-conjugated goat anti-rabbit (1:15.000, Bethyl, cat. 1,704,150) for 1 h at RT. Bands were visualized by Clarity ECL (Bio-Rad, cat. 1,705,060) and the densitometry analysis performed by ImageLab Software (Version 6.1, Bio-Rad). Densitometric values were normalized to Ponceau staining and the relative expression of DAT in *Fmr1-*^*Δ*^*exon 8* rats was reported as fold change on WT controls.

### Statistical analysis

#### Behavioral analysis

Data are expressed as mean ± standard error of the mean (S.E.M.). To assess the effects of the genotype on behavioral parameters, data were analyzed with Student’s t-tests. Sample size (n) is indicated in the figure legends and was based on our previous experiments and power analysis performed with the software G*Power. Potential outliers within each data set were calculated using GraphPad Prism 8 software (Grubbs’ method). A trained observer who was unaware of the treatments assessed all behavioral tests that were scored using the Observer 3.0 software (Noldus Information Technology, NL).

#### DAT imaging analysis

The measured SBR_STR_ values of the two groups were analyzed with Student’s t-tests and expressed as mean ± S.E.M. Data analysis was performed using GraphPad Prism 8 software.

#### Western blot analysis

The statistical differences between the two groups were analyzed with Student’s t-tests and relative values expressed as mean ± S.E.M. Data analysis was performed using GraphPad Prism 8 software.

### Ethical approval

This study was performed and reported in compliance with the ARRIVE guidelines^40, 62^. All applicable Institutional and National guidelines for the care and use of animals were followed. The protocol was approved by the Italian Ministry of Health (Rome, Italy; Authorization N° 849/2020-PR).

## Results

### Behavioral studies. Stereotypic behavior and locomotor activity in ***Fmr1-***^***Δ***^***exon 8*** rats

**Figure 2:**
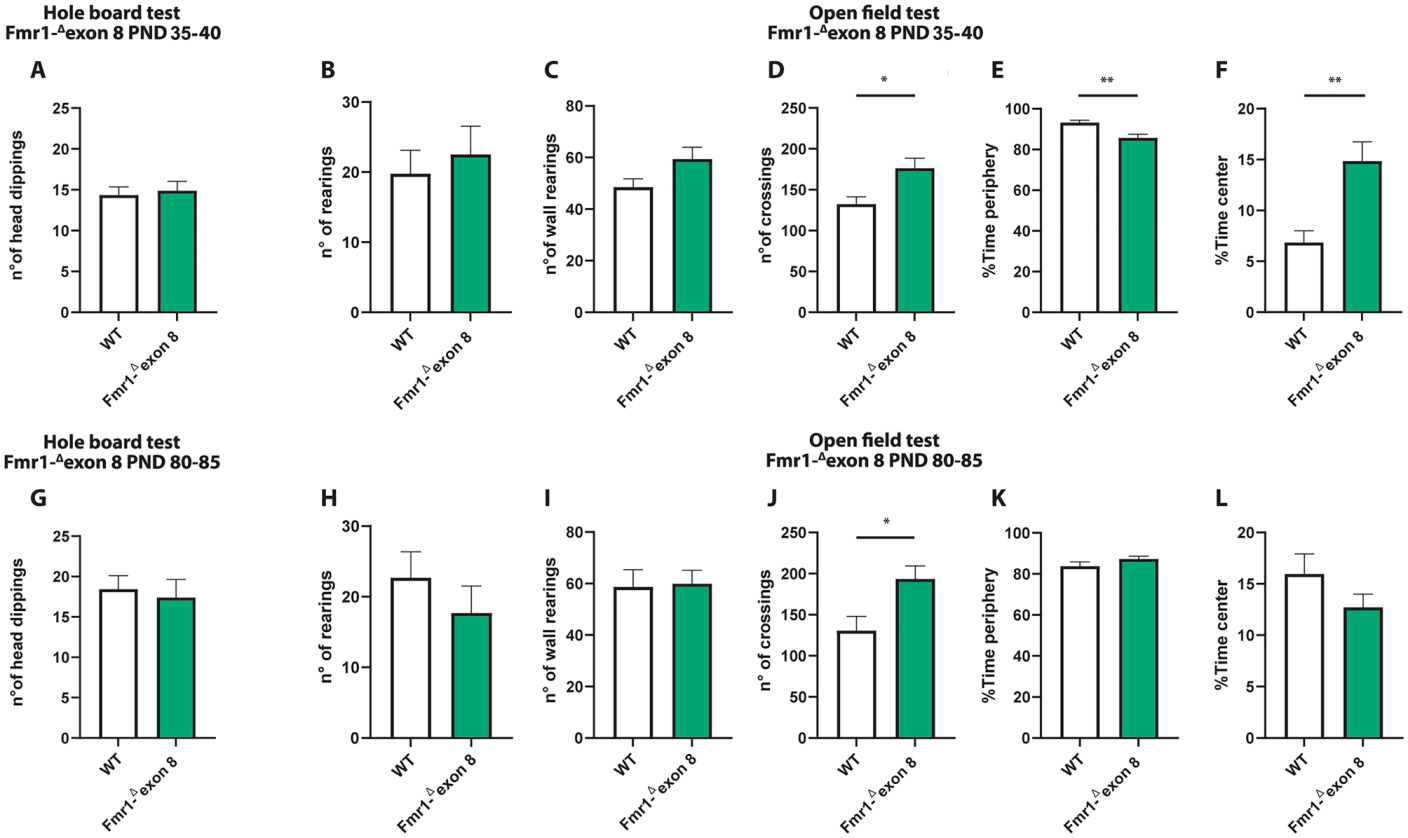
Behavioral studies: Stereotyped behavior and locomotor activity in *Fmr1-*^*Δ*^*exon 8* rats. Both juvenile (**A**) and adult (**G**) *Fmr1-*^*Δ*^*exon 8* rats did not display repetitive behaviors in the hole board test as the number of head dippings did not differ between wild-type (WT) and *Fmr1-*^*Δ*^*exon 8* rats (PND 35-40: WT = 11; *Fmr1-*^*Δ*^*exon 8* = 14; PND 80-85: WT = 18; *Fmr1-*^*Δ*^*exon 8* = 15). Moreover, juvenile and adult *Fmr1-*^*Δ*^*exon 8* rats did not display repetitive behaviors in the open field test as their number of rearings (**B** and **H**) and wall rearings (**C** and **I**) was similar to WT animals. Conversely, *Fmr1-*^*Δ*^*exon 8* rats displayed hyperactivity in the open field test as expressed in the increased number of crossings when compared to WT controls (**D** and **J**). Moreover, juvenile *Fmr1-*^*Δ*^*exon 8* rats spent less time in the periphery of the open field (**E**) and longer time in the center of the arena (**F**) as compared to WT rats. These differences were not observed at adulthood (**K** and **L,** respectively) (PND 35-40: WT = 8; *Fmr1-*^*Δ*^*exon 8* = 8; PND 80-85: WT = 10; *Fmr1-*^*Δ*^*exon 8* = 10). Data represents means ± SEM. *p < 0.05, **p < 0.01 versus WT group (Student’s t-test).

Both juvenile and adult *Fmr1-*^*Δ*^*exon 8* rats did not display repetitive behaviors in the hole board test as the number of head dippings did not differ between WT and *Fmr1-*^*Δ*^*exon 8* rats (PND 35–40: t = 0.34, *p* = n.s, df = 34; PND 80–85: t = 0.38, *p* = n.s, df = 31; Fig. 2A and G). Moreover, both juvenile and adult *Fmr1-*^*Δ*^*exon 8* rats did not display repetitive behaviors in the open field test as the number of rearings (PND 35–40: t = 0.52, *p* = n.s, df = 14; PND 80–85: t = 0.94, *p* = ns, df = 18; Fig. 2 (B and H) and wall rearings (PND 35–40: t = 1.92, *p* = n.s, df = 14; PND 80–85: t = 0.14, *p* = ns, df = 18; Fig. 2C and I) was similar to the WT group.

Conversely, *Fmr1-*^*Δ*^*exon 8* rats displayed hyperactivity in the open field test as they presented an increased number of crossings when compared to their WT controls (PND 35–40: t = 2.92, *p* < 0.05, df = 14; PND 80–85: t = 2.69, *p* < 0.05, df = 18; Fig. 2D and J). Moreover, only juvenile *Fmr1-*^*Δ*^*exon 8* rats spent less time in the periphery of the open field as compared to WT rats (PND 35–40: t = 3.47, *p* < 0.01, df = 14; PND 80–85 t = 1.40, *p* = n.s., df = 18; Fig. 2E and K), and longer time in the central part of the arena (PND 35–40: t = 3.56, *p* < 0.01, df = 14; PND 80–85: t = 1.40, *p* = n.s., df = 18; Fig. 2F and L), suggesting a reduced thigmotaxis in juvenile but not adult *Fmr1-*^*Δ*^*exon 8* rats. To evaluate whether the hyperactivity displayed by *Fmr1-*^*Δ*^*exon 8* rats could be related to an anxious phenotype, we also performed the elevated plus maze test across development. As a result, both juvenile and adult *Fmr1-*^*Δ*^*exon 8* rats did not display anxiety-like behaviors (Supplementary Fig. 1): for instance, no differences were found in the percentage of time spent in the open arms (PND 35–40: t = 0.03, *p* = n.s., df = 33; PND 80–85: t = 0.24, *p* = n.s., df = 34; Supplementary Fig. 1 (A and E)), in the percentage of open arm entries (PND 35–40: t = 0.43, *p* = n.s., df = 33; PND 80–85: t = 0.20, *p* = n.s., df = 34; Supplementary Fig. 1 (B and F)), and in the frequency of head-dippings (PND 35–40: t = 0.77, *p* = n.s., df = 33; PND 80–85: t = 0.20, *p* = n.s., df = 34; Supplementary Fig. 1 (C and G)). Conversely, both juvenile and adult *Fmr1-*^*Δ*^*exon 8* rats displayed hyperactivity in the elevated plus maze test as they showed an increased number of total arm entries when compared to their WT controls (PND 35–40: t = 3.64, *p* < 0.001, df = 33; PND 80–85: t = 2.87, *p* < 0.01, df = 34; Supplementary Fig. 1 (D and H)). This further confirms the hyperlocomotion displayed by *Fmr1-*^*Δ*^*exon 8* rats across development.

### Neuroimaging studies

**Figure 3:**
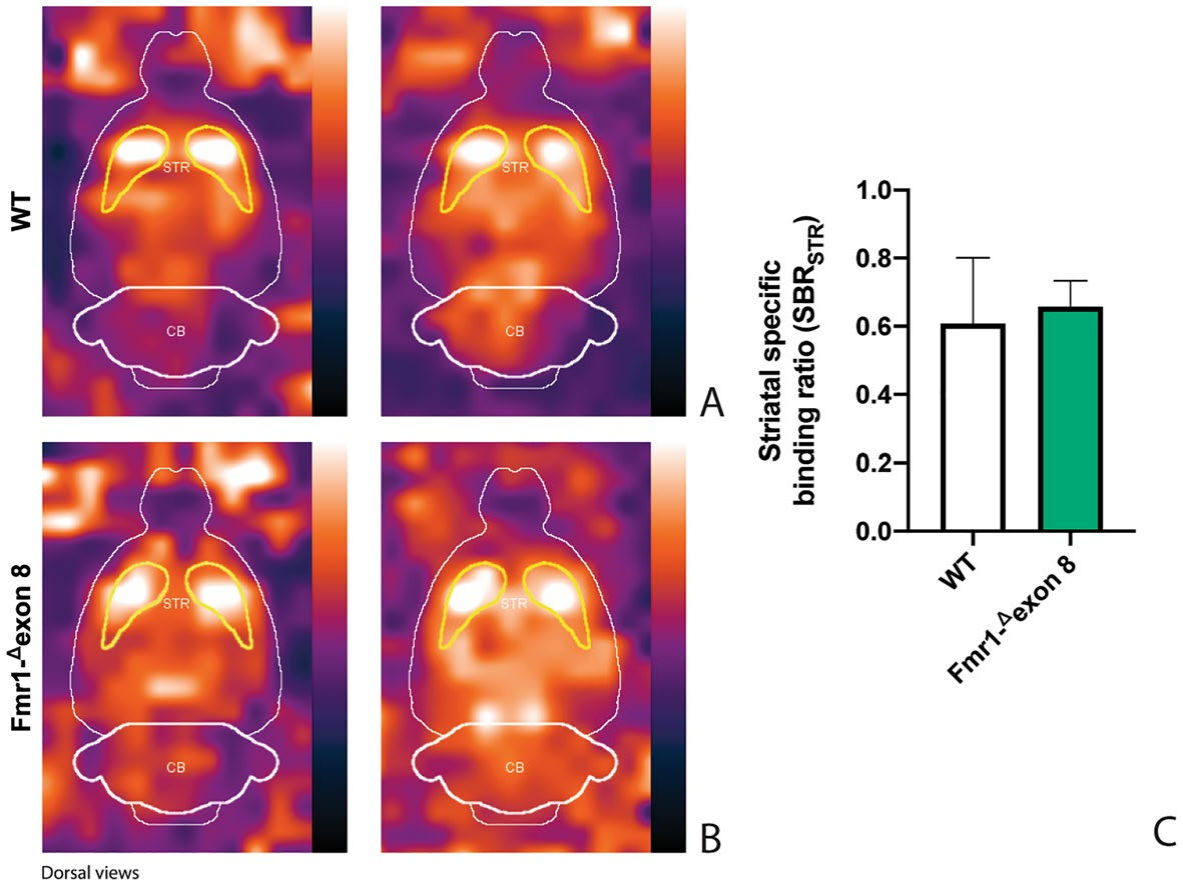
DAT binding. The estimates for the binding potentials were evaluated through the equilibrium ratios of the distribution areas of the specifically (striatum) and non-specifically (cerebellum) bound and striatal specific binding ratios (SBR_STR_) for WT and *Fmr1-*^*Δ*^*exon 8* rats. (**A**) [^123^I]FP-CIT binding to striatal DAT in two WT adult rats. (**B**) [^123^I]FP-CIT binding to striatal DAT in two *Fmr1-*^*Δ*^*exon 8* adult rats. The striatal specific DAT binding ratio is shown in (**C**) (WT = 5; *Fmr1-*^*Δ*^*exon 8* = 5). Data represents means ± SEM (Student’s t-test).

**Figure 4:**
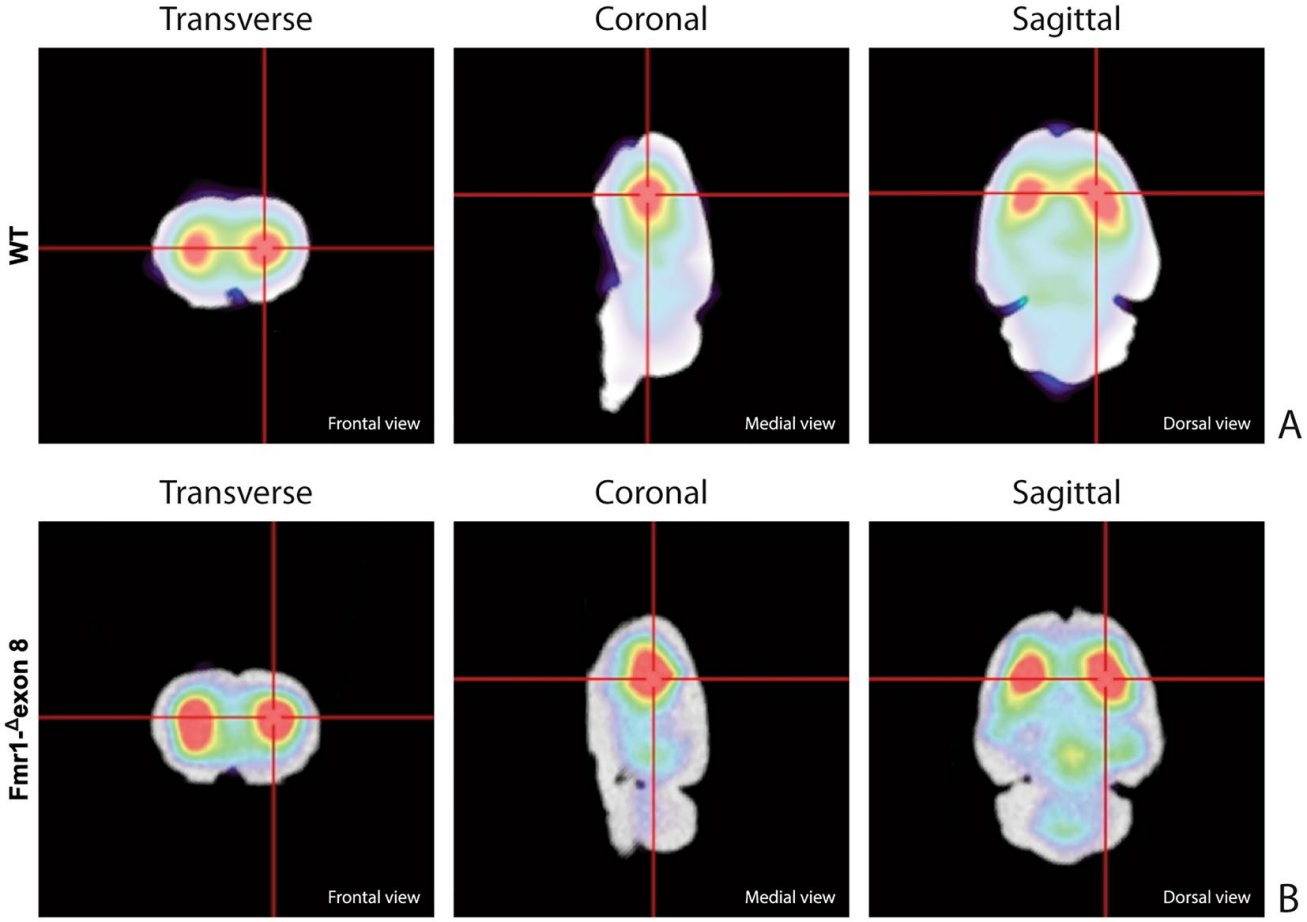
SPECT OSEM reconstruction of the distribution of [^123^I]FP-CIT uptake in the rat brain. (**A**) Orthogonal views in WT adult rats. (**B**) Orthogonal views in *Fmr1-*^*Δ*^*exon 8* adult rats.

**Figure 5:**
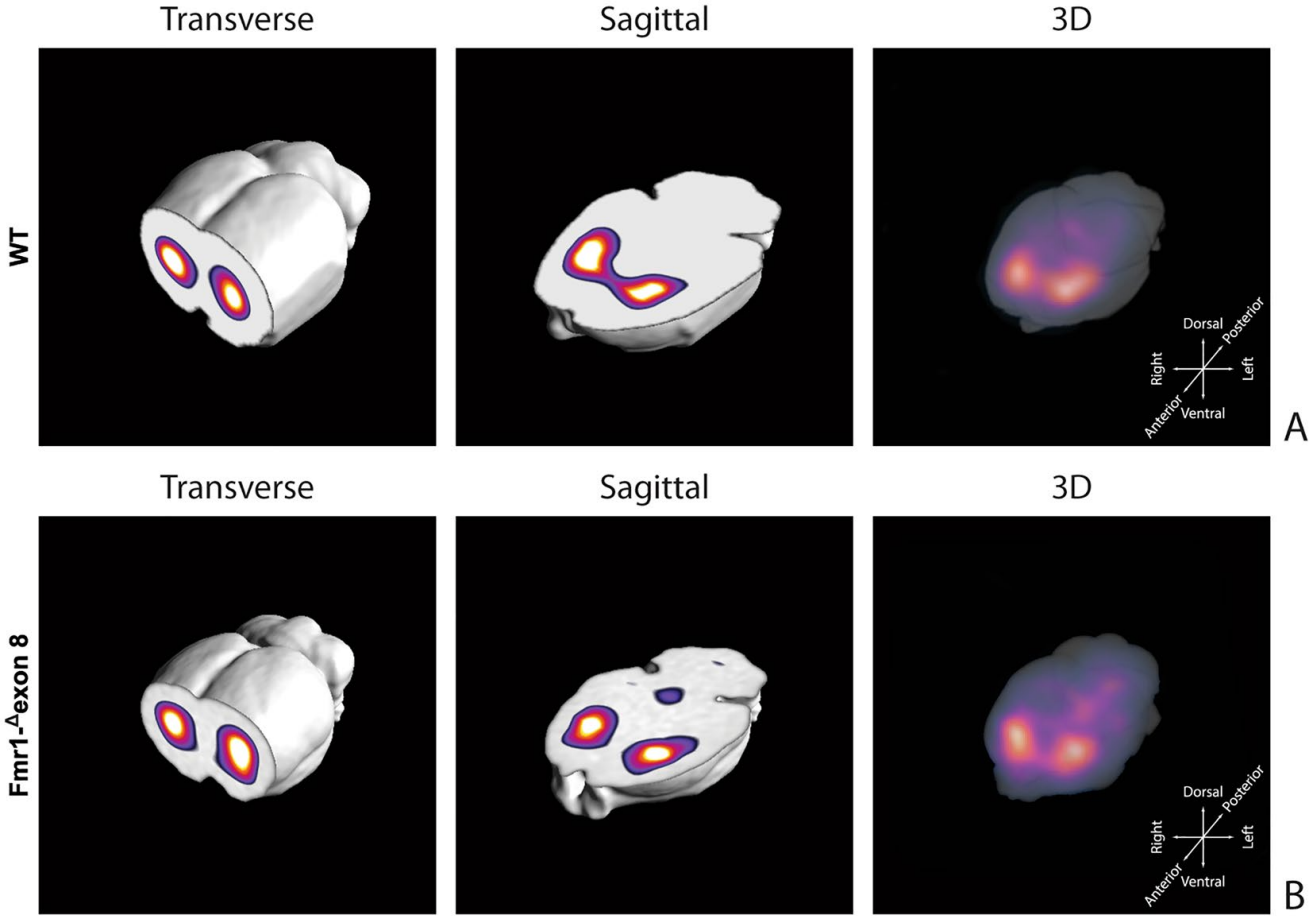
Rat brain volumetric rendering applying the 2-step trans-axial SSR technique. (**A**) [^123^I]FP-CIT 3D uptake in WT adult rats. (**B**) [^123^I]FP-CIT 3D uptake in *Fmr1-*^*Δ*^*exon 8* adult rats.

Figure 3 shows the characteristic planar images of [^123^I]FP-CIT uptake of two WT rats (panel A) and two *Fmr1-*^*Δ*^*exon 8* (panel B) rats, respectively. Radioactivity accumulations are clearly visible in the striatum. Moreover, the image also highlights the ROIs related to both striatum and cerebellum for each experimental group. SBR_STR_ analysis reveals that in the WT group the striatal specific binding ratio was 0.608 ± 0.087 (mean ± S.E.M.), whereas for the *Fmr1-*^*Δ*^*exon 8* rats the value was 0.658 ± 0.034 (mean ± S.E.M.). As a result, the unpaired t-test revealed no significant between-group differences (t = 0.539, *p* = 0.604, df = 8, Fig. 3C). Since the images also showed enhanced DAT levels in the midbrain, its specific binding ratio have been assessed. Namely, the midbrain specific binding ratio was 0.332 ± 0.135 (mean ± S.E.M.) in the WT group, and 0.403 ± 0.034 (mean ± S.E.M.) in *Fmr1-*^*Δ*^*exon 8* rats. As with striatal uptake, the unpaired t-test revealed no significant between-group differences (t = 0.716, *p* = 0.548, df = 2). An example of the characteristic distribution of DAT in the striatum in both experimental groups, WT (top) and *Fmr1-*^*Δ*^*exon 8* (bottom), is also shown through an orthogonal view of the SSR SPECT OSEM reconstruction restricted to CT brain data (Fig. 4). Moreover, the DAT binding distribution with respect to the correlated CT brain volume obtained from a 3D rendering of the dataset is shown in Fig. 5 (WT on top and *Fmr1-*^*Δ*^*exon 8* on bottom images). An initial visual analysis of the images for both groups does not show a difference in the number and extent of radiopharmaceutical uptake, which is overall normal for both nigrostriatal systems. This assessment is corroborated by semi-quantitative SBR_STR_ analysis, which reveals no substantial differences between the two groups.

### Immunofluorescence and immunohistochemistry analysis

**Figure 6:**
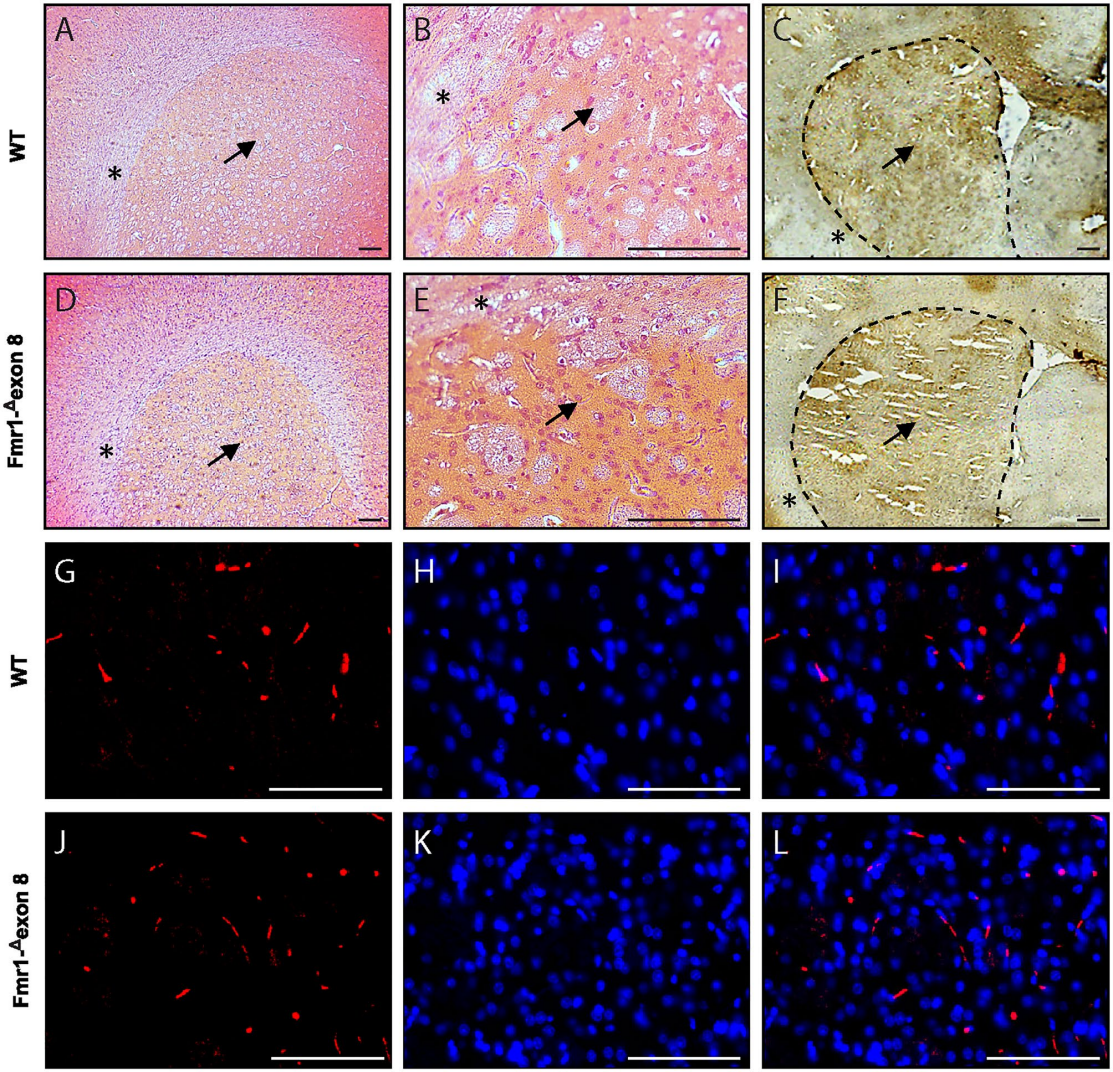
Histological and immunostaining assays. Microphotographs of hematoxylin and eosin staining of dorsal striatum of WT (**A** and **B**) and *Fmr1-*^*Δ*^*exon 8* (**D** and **E**) rats at lower magnification (6X, **A** and **D**) and higher magnification (40 × , **B** and **E**). Representative DAT immunohistochemistry in the dorsal striatum of adult WT (**C**) and *Fmr1-*^*Δ*^*exon 8* (**F**) rats. The dorsal striatum and surrounding corpus callosum are indicated with a black arrow and asterisks, respectively, both in histological and immunohistochemistry images. Scale-bar: 100 µm. DAT immunofluorescence and DAPI nuclear staining in the dorsal striatum of adult WT (**G–I**) and *Fmr1-*^*Δ*^*exon 8* (**J–L**). Red DAT (**G** and **J**); blue DAPI (**H** and **K**); merge (**I** and **L**). Scale-bar: 100 µm.

Hematoxylin and eosin (H&E) staining was performed to visualize possible gross histopathological changes in the dorsal striatum of *Fmr1-*^*Δ*^*exon 8* rats as compared to their WT controls. We found that the structure of the *Fmr1-*^*Δ*^*exon 8* and WT rat brain tissues analyzed by H&E histology (Fig. 6A and B for WT rats, D and E for *Fmr1-*^*Δ*^*exon 8* rats) was comparable at the level of the dorsal striatum (marked with a black arrow), suggesting no changes in the gross tissue organization of this brain region between *Fmr1-*^*Δ*^*exon 8* and WT rats. Moreover, immunohistochemical staining relative to DAT expression (highlighted as a dark brown dye) in the dorsal striatum (Fig. 6C for WT and F for *Fmr1-*^*Δ*^*exon 8* rats) showed no qualitative differences between genotypes as also confirmed by immunofluorescence in the striatal DA axonal projections (Fig. 6G, H and I for WT and J, K and L for *Fmr1-*^*Δ*^*exon 8* rats). These findings strengthen our neuroimaging results revealing no differences in DAT between genotypes.

### Western blot analysis

**Figure 7:**
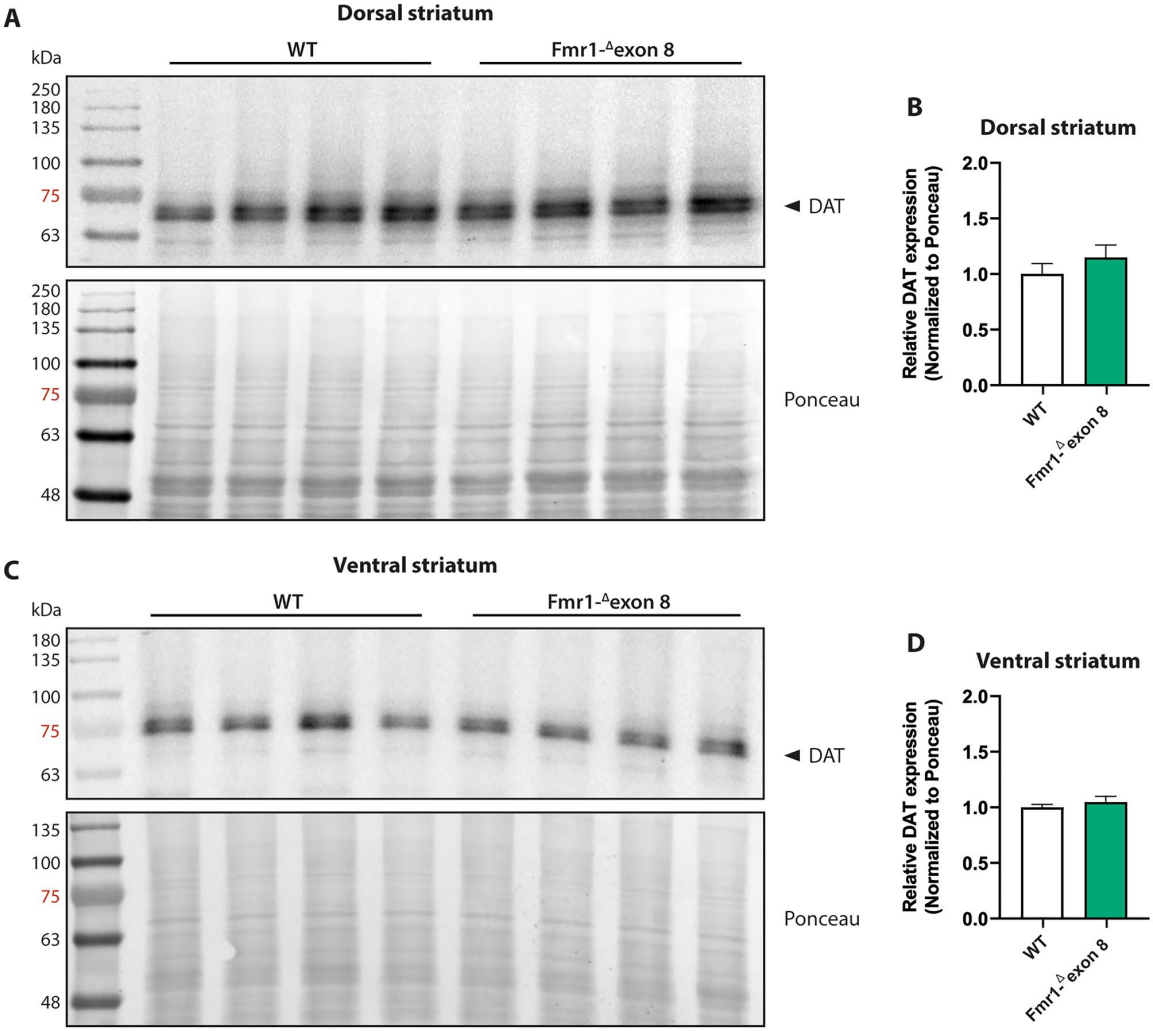
Western blot analysis. Representative western blot images of DAT in the dorsal (**A**) and ventral (**C**) striatum lysates from adult WT and *Fmr1-*^*Δ*^*exon 8* rats. Quantitative analysis of DAT protein levels in the dorsal striatum (**B**) and ventral striatum (**D**) from adult WT and *Fmr1-*^*Δ*^*exon 8* rats (WT = 4; *Fmr1-*^*Δ*^*exon 8* = 4). Densitometric values of *Fmr1-*^*Δ*^*exon 8* rats were normalized on Ponceau staining and expressed as relative fold change on WT animals. Data represents means ± SEM (Student’s t-test).

To estimate the protein expression level of DAT in the striatum of *Fmr1-*^*Δ*^*exon 8* rats, we performed a western blotting analysis in both the dorsal (Fig. 7A and B) and ventral striatum (Fig. 7C and D). In line with SPECT and immunohistochemical findings, the DAT protein levels in both the dorsal (t = 1.02, df = 6, *p* = n.s.) and ventral (t = 0.81; df = 6; *p* = n.s.) striatum did not differ between WT and *Fmr1-*^*Δ*^*exon 8* animals. Overall, these data confirm that FMRP deficiency does not induce a dysregulation in striatal DAT expression and that no regional (dorsal vs. ventral striatum) differences in DAT expression underlie the dysfunctional motor behavior displayed by *Fmr1-*^*Δ*^*exon 8* rats. Original uncropped membranes are shown in Supplementary Fig. 2 (A and B).

## Discussion

In the present work, we combined behavioral, biochemical and in vivo neuroimaging analyses to investigate the role of striatal DAT expression in the dysfunctional motor behavior of *Fmr1-*^*Δ*^*exon 8* rats, a genetic animal model of ASD and a rat model of FXS.

Since FXS patients often show hyperactivity and repetitive patterns of behavior ^3^, we here tested *Fmr1-*^*Δ*^*exon 8* rats in two behavioral tasks aimed to reveal possible stereotyped/repetitive behaviors and exploratory/locomotor alterations: the hole board and the open field tests. Our results showed that both juvenile and adult *Fmr1-*^*Δ*^*exon 8* rats displayed hyperactivity in the open field test expressed as increased number of crossings while performing the task. Juvenile *Fmr1-*^*Δ*^*exon 8* rats also spent less time in the peripheral part of the open field (that is, close to the walls of the open field). To evaluate whether the hyperactivity displayed by *Fmr1-*^*Δ*^*exon 8* rats could be related to changes in anxiety-like behaviors, we also performed the elevated plus maze test. Both juvenile and adult *Fmr1-*^*Δ*^*exon 8* rats did not display anxiety-like behaviors in this task. Conversely, both juvenile and adult *Fmr1-*^*Δ*^*exon 8* rats showed an increased number of total entries in the arms of the elevated plus maze when compared to WT controls, thus confirming the hyperlocomotion displayed by *Fmr1-*^*Δ*^*exon 8* rats across development. In line with these results, the extensively characterized Fmr1-KO mouse model of FXS has been shown to display motor alterations and hyperactivity^63–67^, although normal locomotor activity has also been also reported in rat models of FXS^68,69^. Conversely, we found that *Fmr1-*^*Δ*^*exon 8* rats did not show stereotypic/repetitive behaviors in the hole-board test, as the number of head dippings did not differ from their WT controls. Depending on the animal strain and the behavioral task used, some studies^66,70,71^ but not others^63,69,72^ reported stereotyped behaviors in both rat and mouse models of FXS. Thus, we cannot exclude that *Fmr1-*^*Δ*^*exon 8* rats would show repetitive patterns of behavior if different tasks or experimental protocols were used.

Evidence for an involvement of dopaminergic neurotransmission in ASD arises from neuroimaging, genetic and pharmacological studies in individuals with autism but also from preclinical research performed in rodent models of ASD^73^. Over the years, techniques as SPECT/PET have been employed enabling imaging of the dopaminergic system in different psychiatric and neurological disorders. Such methods are often based on the assessment of DAT density as a marker for dopaminergic neuron integrity^55,74–76^. Generally, a radiolabel such as ^123^I-FP-CIT (DaTSCAN) is used since it is able to bind with high affinity to the presynaptic DAT located on axon terminals in the striatum. In the clinical practice, SPECT with ^123^I-FP-CIT can be reported as normal or abnormal through the semi-quantitative measurement of the ^123^I-FP-CIT signal uptake in the DAT^55^. Therefore, imaging with specific DA-related tracers represents a valuable tool to evaluate the status of presynaptic nigrostriatal terminals. In particular, the radiotracer DaTSCAN has become part of the diagnostic guidelines for α-synucleinopathies (e.g., Parkinsonian Syndromes, multiple system atrophy, and dementia with Lewy bodies), being approved by the most competent international authorities (i.e., FDA and EMA)^77,78^. From a methodological point of view, SPECT images might be exploited to determine to what extent this tracer is accumulated in the striatum compared to the background signal. As a result, reduced DAT striatal binding is therefore suggested to depict reduced DAT availability which in turn reflects striatal dopaminergic deficit^79^. PET imaging showed increased DAT binding in the orbitofrontal cortex of high-functioning adults with ASD^80^, although a SPECT study in children with autism showed no changes in DAT binding^81^. Interestingly, a study that examined striatal dopamine functioning during monetary incentive processing in ASD patients and controls using simultaneous PET and fMRI reported impaired phasic DA release to rewards in the striatum of patients with autism^82^. While these clinical findings support the involvement of functional changes in dopaminergic neurotransmission in ASD, differences in experimental procedures and heterogeneity in the patient populations also lead to conflicting results across studies and warrant further investigation.

Here, we took advantage from a recently developed innovative SPECT system for imaging in laboratory rodents^39^ to investigate whether the hyperactivity displayed by *Fmr1-*^*Δ*^*exon 8* rats was accompanied by changes in striatal DAT availability. By performing scintigraphic SPECT analyses in vivo using ^123^I-FP-CIT as radiolabel, we found comparable DAT availability in the striatum of *Fmr1-*^*Δ*^*exon 8* rats and WT controls, suggesting that no changes in striatal DAT expression occurred in *Fmr1-*^*Δ*^*exon 8* rats. Based on these results, it is tempting to speculate that the altered locomotor activity (as expressed by the increased number of crossings in the open field and the increased total entries in the elevated plus maze) we consistently observed in *Fmr1-*^*Δ*^*exon 8* rats along development might not be attributable to an alteration of striatal DAT availability. This evidence is also corroborated by immunohistochemistry and immunofluorescence analyses, since our results showed no difference in DAT expression between WT and *Fmr1-*^*Δ*^*exon 8* rats in the dorsal striatum. To evaluate for a possible different contribution of the two distinct districts of the striatum (i.e., dorsal and ventral), we performed Western blot analysis of DAT protein levels: notably, no significant differences were found between genotypes, indicating that DAT protein levels were preserved in both dorsal and ventral striatum of *Fmr1-*^*Δ*^*exon 8* rats. Despite these consistent results, we cannot exclude that pharmacological manipulation of the dopaminergic system (e.g., administration of D_1_ and D_2_ agonist/antagonist, cocaine, or blockers of other transporters) could have an impact on the altered locomotor activity shown by *Fmr1-*^*Δ*^*exon 8* rats, as previously reported in Fmr1 KO mice^83–86^ and DAT-KO mice and rats^24,87,88^. Moreover, investigating possible DA-independent mechanisms that may drive hyperactivity in *Fmr1-*^*Δ*^*exon 8* rats also remains an intriguing point that deserves further investigation. For instance, the pharmacological targeting of serotonin^89,90^, GABA^91,92^, BDNF^93,94^, acetylcholine^95^ pathways (to mention a few) has been demonstrated to ameliorate abnormal locomotor behaviors in Fmr1-KO mice.

Despite dopaminergic aberrations have been extensively documented in Fmr1-KO mice^63,96–99^, preclinical data on the striatal DAT activity remain controversial, with some studies reporting a decreased striatal DAT expression^97^, whereas others (including the present results) showing no change in DAT function^99^. Based on these (apparent) contradictory findings, we should consider the hypothesis that specific changes in DA signaling may differentially contribute to ASD pathophysiology and consequently may (not) account for the full spectrum of ASD-related behavioral manifestations^73^. For instance, it has been shown that activation of D_2_ expressing neurons in the ventral striatum reduced running and locomotion in mice, while D_2_ expressing neuron inhibition had opposite effects^100^; moreover, FMRP seems to be involved in D_1_ -mediated neuroplasticity in the prefrontal cortex^101–103^. Besides, DA receptors are differentially integrated in cortical circuit components subserving distinct aspects of cognitive control, including relaying motor commands^104^. This will undoubtedly contribute to clarify the cellular (D_1_ vs. D_2_) and regional (dorsal vs. ventral striatum, prefrontal cortex, cerebellum) specificity of DA pathways in mediating motor dysfunctions in *Fmr1-*^*Δ*^*exon 8* rats.

As referring to the SPECT methodology, it is important to clarify that FP-CIT is not a substrate for the transporter, hence imaging analysis only provides DAT expression. Accordingly, it is possible that alterations in the dopaminergic system contribute to the observed hyperactivity phenotype through one of the following mechanisms: (i) altered trafficking or catalytic activity of DAT; (ii) altered synthesis, packaging, or release of DA; (iii) altered sensitivity of DA receptors^105^. These hypotheses warrant further investigation in a follow-up of this study.

Overall, our results showed that *Fmr1-*^*Δ*^*exon 8* rats displayed hyperactivity in the open field and in the elevated plus maze tests, in the absence of repetitive behaviors in the hole board test, with no changes in striatal DAT availability as assessed by in vivo SPECT imaging and Western blot experiments. This study supports a preservation of striatal DAT availability following FMR1 deletion in rats and confirms that in vivo SPECT imaging paralleled by behavioral observation represents a useful tool to non-invasively investigate variations in neurotransmitter activity in neurodevelopmental disorders. Since sex-dependent differences in preclinical models of ASD have been documented^106^, the inclusion of both male and female animals should be considered in future studies.

## Supplementary Information

Supplementary Figure 1.

Supplementary Figure 2.

## Data Availability

The datasets used and/or analysed during the current study available from the corresponding author on reasonable request.
