# Supplementary figures and images for: FMR1 deletion in rats induces hyperactivity with no changes in striatal dopamine transporter availability
Source: Sci Rep. 2022 Dec 29;12:22535. doi: 10.1038/s41598-022-26986-2 (PMC9800572; doi:10.1038/s41598-022-26986-2)

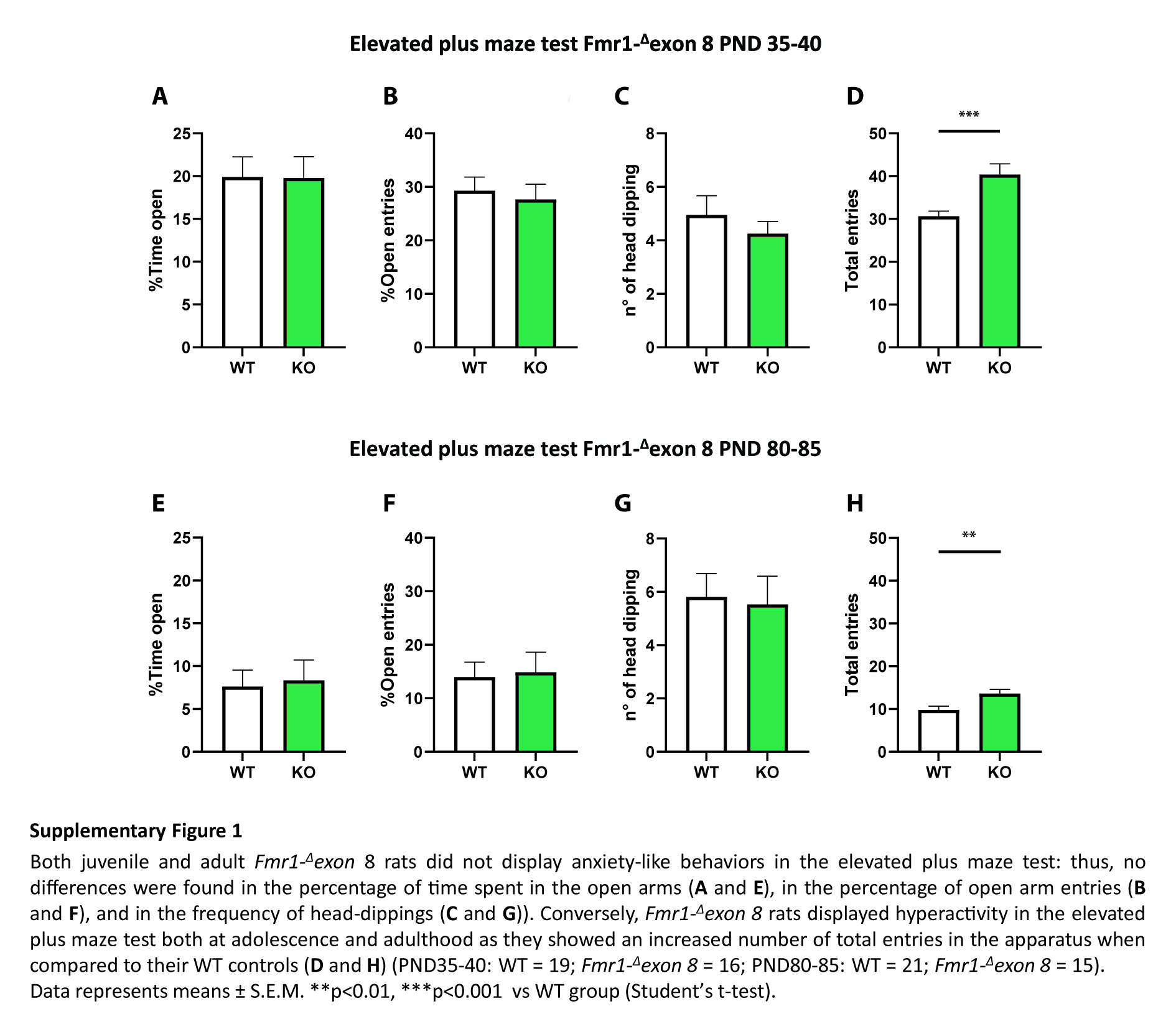

Supplement: Supplementary file 1 — Supplementary Figure 1. [file 41598_2022_26986_MOESM1_ESM.tif]

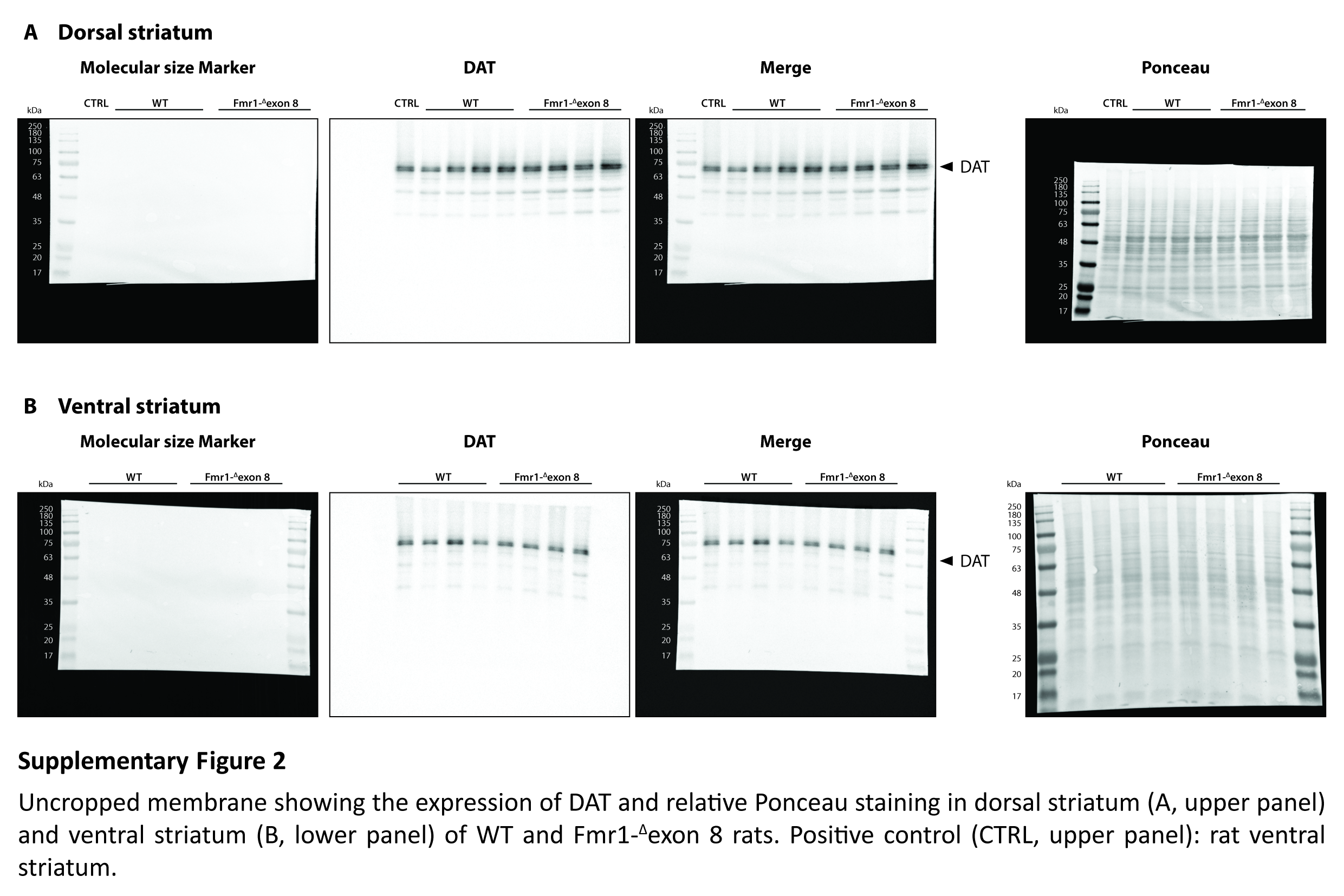

Supplement: Supplementary file 2 — Supplementary Figure 2. [file 41598_2022_26986_MOESM2_ESM.tif]
